# Production of ethanol from winter barley by the EDGE (enhanced dry grind enzymatic) process

**DOI:** 10.1186/1754-6834-3-8

**Published:** 2010-04-28

**Authors:** NP Nghiem, KB Hicks, DB Johnston, G Senske, M Kurantz, M Li, J Shetty, G Konieczny-Janda

**Affiliations:** 1 Eastern Regional Research Center, Agricultural Research Service, US Department of Agriculture, Wyndmoor, PA 19038, USA; 2 Genencor, Danisco Division, Palo Alto, CA 94304, USA

## Abstract

**Background:**

US legislation requires the use of advanced biofuels to be made from non-food feedstocks. However, commercialization of lignocellulosic ethanol technology is more complex than expected and is therefore running behind schedule. This is creating a demand for non-food, but more easily converted, starch-based feedstocks other than corn that can fill the gap until the second generation technologies are commercially viable. Winter barley is such a feedstock but its mash has very high viscosity due to its high content of β-glucans. This fact, along with a lower starch content than corn, makes ethanol production at the commercial scale a real challenge.

**Results:**

A new fermentation process for ethanol production from Thoroughbred, a winter barley variety with a high starch content, was developed. The new process was designated the EDGE (enhanced dry grind enzymatic) process. In this process, in addition to the normal starch-converting enzymes, two accessory enzymes were used to solve the β-glucan problem. First, β-glucanases were used to hydrolyze the β-glucans to oligomeric fractions, thus significantly reducing the viscosity to allow good mixing for the distribution of the yeast and nutrients. Next, β-glucosidase was used to complete the β-glucan hydrolysis and to generate glucose, which was subsequently fermented in order to produce additional ethanol. While β-glucanases have been previously used to improve barley ethanol production by lowering viscosity, this is the first full report on the benefits of adding β-glucosidases to increase the ethanol yield.

**Conclusions:**

In the EDGE process, 30% of total dry solids could be used to produce 15% v/v ethanol. Under optimum conditions an ethanol yield of 402 L/MT (dry basis) or 2.17 gallons/53 lb bushel of barley with 15% moisture was achieved. The distillers dried grains with solubles (DDGS) co-product had extremely low β-glucan (below 0.2%) making it suitable for use in both ruminant and mono-gastric animal feeds.

## Background

There is an increasing interest in alternative liquid fuels, and in particular ethanol, throughout the world. In the USA the goal is to produce 36 billion gallons of renewable fuels by 2022, of which 22 billion gallons are 'advanced biofuels' made from non-corn feedstocks [1]. Ethanol production from corn in the USA has more than quadrupled from 1.6 billion gallons per year in 2000 to 9 billion gallons per year in 2008 [2] and it is expected to continue to increase. However, it has been estimated that the maximum quantity of ethanol that can be produced from corn in the USA without negatively impacting on the feed and food markets is only about 15 to 16 billion gallons per year [3], which is well below the national goal. Thus, production of ethanol from renewable feedstocks other than corn is needed. Lignocellulosic biomass can help to meet the stated goal but the technology is not ready for commercialization and further development is still required [4]. The key issue in lignocellulosic biomass utilization is the difficulty in converting the carbohydrate fractions to fermentable sugars at high yield and in an economical way [5]. On the other hand, starch, which is the main carbohydrate in corn and other grains, can be readily hydrolyzed to glucose at high yield by commercially available and low-cost enzymes [6]. It is, therefore, a good idea to develop processes for producing ethanol from starch feedstocks other than corn in order to supplement the corn ethanol production while waiting for the lignocellulosic biomass ethanol technology to be ready for commercialization. A starch-based feedstock, which can be readily fermented to ethanol and sustainably produced, is therefore of great interest. Barley qualifies as an ethanol feedstock. It can be grown outside the 'corn belt' [7] and also has the potential of adding about 2 billion gallons per year of ethanol to North America's annual output [8]. On the East coast, and in other regions of the country with mild winters, barley is grown as a winter crop and acts as a ground cover to prevent excess nutrients from leaching into watersheds and sensitive areas such as the Chesapeake Bay [9]. Harvesting winter barley in late May/early June allows for the production of a full soybean crop afterwards in the same crop year. Following the next year with corn and then winter barley, allows a 2-year, three-crop rotation. This process results in more grain being produced on the same acreage with less nutrient loss to sensitive waterways, which is a win/win situation for both renewable fuels and the environment [10,11]. As the winter barley is grown on winter fallow land that would not otherwise be in use, it requires no new land and does not interfere with food production, thus avoiding any potential indirect land use changes.

One of the challenges of using barley in fuel ethanol fermentation is the presence of mixed linkage (1,3)(1,4)-β-D-glucans in the grains. These polymers constitute the largest non-starch polysaccharide component of the endosperm cell wall and account for approximately 3.0% to 4.5% of the total grain weight [12]. During the preparation of the mash, β-glucans become soluble in water and cause the viscosity to increase considerably. In fuel ethanol fermentation, where total solid contents of 30% are used, the extremely high viscosity of the mash severely impedes mixing which, in turn, will negatively affect distribution of the added yeast and nutrients. The viscosity problem could be partially resolved by the addition of a commercial β-glucanase obtained from *Aspergillus niger *to the barley mash. However, the addition of this enzyme did not result in higher ethanol yield [13]. When barley β-glucans were hydrolyzed with β-glucanase from the fungus *Talaromyces emersonii *only small quantities of glucose were generated with the rest of the hydrolysis products being oligosaccharides which had a degree of polymerization (DP) from 2 - 5 [14]. The β-glucanase used in the early investigation in which no improvement on ethanol yield was observed [13] also most probably hydrolyzed the barley β-glucans to primarily glucose oligosaccharides that are not fermentable by the ethanologenic yeast *Saccharomyces cerevisiae *[15].

The presence of β-glucans in feeds used for poultry also displayed anti-nutritional effects [16]. Thus, high levels of β-glucans in DDGS may make this fuel ethanol co-product unsuitable for use in feeds for monogastric animals.

In this paper, we report on the development of a shake-flask scale fermentation process for production of ethanol from Thoroughbred, which is an improved version of hulled winter barley with significantly higher starch contents and test weights (lb per bushel) compared to regular feed barley and is available in the USA [17]. This process is designated the EDGE (enhanced dried grind enzymatic) process because it involves the use of β-glucanases to effectively hydrolyze the β-glucans in the barley grains plus a β-glucosidase to subsequently hydrolyze the products of the first hydrolysis to glucose, which eventually results in increased ethanol production. In addition, the use of the β-glucanases also results in a DDGS with an extremely low β-glucan content, which makes this co-product suitable for use in feed for all animals.

A fermentation process has been developed by Danisco (Copenhagen, Denmark) for the production of ethanol from barley. In this process, ground barley is first mixed with water at 28%-30% dry solids to make a slurry. Three enzymes are added, which include OPTIMASH™ BG (a β-glucanase) at 0.13 kg/ton solids, OPTIMASH™ TBG (a thermostable β-glucanase) at 0.06 kg/ton solids and SPEZYME^® ^Xtra (a thermostable α-amylase) at 0.30 kg/ton solids. The slurry is adjusted to pH 5.2 and maintained at 60°C for 1 h. Next, liquefaction of the starch is performed by raising the temperature to 85°-90°C and maintained for 3 h. In the last step, which is a simultaneous saccharification and fermentation (SSF), the pH is adjusted to 3.8-4.2 and the following components are added: urea at 400 ppm final concentration; FERMENZYME^® ^L-400 (a glucoamylase) at 0.65 kg/ton solids; OPTIMASH™ TBG at 0.05 kg/ton solids; and rehydrated dry yeast. The SSF is performed at 32°C for 55-60 h. In this paper we report on: (a) the shake flask-scale optimization of the Danisco process, which is referred to as the base-line process, for the production of ethanol from a particular batch of Thoroughbred winter barley; and (b) the development of an improved shake flask-scale process for the production of ethanol from that batch of Thoroughbred. The key steps of the new process are the same as those used in the base-line process, except that in the new process, in addition to the β-glucanases that are added to reduce the mash viscosity, another enzyme (β-glucosidase) is added to convert the non-fermentable oligosaccharides, which are formed upon enzymatic hydrolysis of β-glucans, to glucose, which is readily fermentable by *S. cerevisiae*. The availability of additional fermentable substrate is expected to result in higher ethanol yields using the new process. The optimum conditions determined for ethanol production using this particular batch of Thoroughbred winter barley by the shake flask-scale EDGE process also are presented.

## Methods

### Materials

Thoroughbred winter hulled barley, originally developed at Virginia Polytechnic Institute and State University, was grown by and obtained from the Virginia Foundation Seed Center, Virginia, USA in 2005. Upon receipt, the grain was placed in a freezer for approximately 3 days in order to eliminate any insects. The barley was subsequently stored in a low humidity room at ambient temperature (18°-24°C) and relative humidity below 25% until used. Four 50 lb bags from the same lot number were thoroughly mixed for 2 min using a tumbling dryer, operated without heat or vacuum. After mixing and sampling the grain was divided equally into four plastic pails containing approximately 50 lb of barley in each pail for storage. The test weight of the aforementioned barley was determined to be 52.9 lb per bushel. The composition of the barley was determined and the results are summarized in Table 1. The methods used for the compositional analysis are described in the analytical section.

**Table 1:** Composition of Thoroughbred barley.

Component	Quantity (%)*
Moisture (whole kernels)	8.09 ± 0.03

Oil†	1.92 ± 0.06

Starch†	59.89 ± 1.20

Protein†	7.60 ± 0.03

B - Glucan†	3.90 ± 0.05

Acid detergent fibre†	5.47 ± 0.21

Neutral detergent fibre†	17.22 ± 1.05

Crude fibre†	4.66 ± 0.12

*Average of three determinations.

† Dry basis.

All of the enzymes, which included SPEZYME^® ^XTRA (thermostable α-amylase), OPTIMASH™ BG (β-glucanase), OPTIMASH™ TBG (thermostable β-glucanase), FERMENZYME^® ^L-400 (glucoamylase/protease mix) and a developmental β-glucosidase, were provided by Genencor International (a Danisco division, New York, USA). The enzymes were kept refrigerated at 4°C.

Active Dry Ethanol Red was provided by Lesaffre Yeast Corporation (Wisconsin, USA). The dry yeast powder was kept refrigerated at 4°C.

All chemicals were of reagent grades and purchased from Sigma-Aldrich (Missouri, USA).

### Base-line ethanol production

Barley was ground in a Wiley mill fitted with either 1 mm or 2 mm screen. The mash for ethanol fermentation was prepared in batches of 1000 g total weight. First, the moisture content of the ground barley was determined using the method described in the analytical section below. Then the quantity of ground barley equivalent to 300 g dry solids was obtained and added to a 2 L beaker containing de-ionized water needed to make a total weight of 1000 g. The slurry was stirred with a mechanical agitator. The pH was adjusted to 5.2 with 2 M sulphuric acid and three enzymes were added, which included SPEZYME^® ^XTRA, added at 81.2 μL (0.30 kg/ton solids), OPTIMASH™ BG, added at 35.5 μL (0.13 kg/ton dry solids) and OPTIMASH™ TBG, added at 16.4 μL (0.06 kg/ton dry solids). The mash was heated on a hot plate and the heating rate was adjusted to maintain the desired temperatures. The mash temperature was maintained at 60°C for 1 h (pre-liquefaction) and then at 90°C for 3 h (liquefaction). During this time, small amounts of de-ionized water were intermittently added to compensate for the evaporation loss. At the end of the starch liquefaction, the beaker was cooled in a water bath. When the temperature of the mash dropped to about 40°C the beaker was weighed and de-ionized water was added to bring the total weight back to 1000 g. The mash was stirred and its pH adjusted to 3.8-4.0 with 2 M sulphuric acid. A glucoamylase plus protease mixture, FERMENZYME^® ^L-400, was added at 177 μL (0.65 kg/MT dry solids) together with OPTIMASH™ BG at 13.5 μL (0.05 kg/MT dry solids) and urea (0.4 g). The main purpose of the OPTIMASH™ BG addition to the cooked mash was to complete the solubilization and liquefaction of the remaining β-glucans that were not liquefied in the pre-liquefaction and liquefaction steps. The enzyme dosages described above were those recommended by the manufacturer. The pre-liquefaction and liquefaction time were the same as those used in the process developed by Danisco (the base-line process). Stirring of the mash was continued for 20 min to ensure the complete dissolution of urea and a uniform distribution of the enzymes. The mash was then dispensed into 250-mL flasks at 150 g/flask. The active dry yeast was rehydrated by addition of 2.5 g to 50 mL de-ionized water and stirred for 30 min. The yeast slurry was added to the flasks at 0.75 mL/flask. The initial viable yeast count was about 2 × 10^7^/g dry solids or 5 × 10^6^/g of mash. The flasks were capped with rubber stoppers which had an 18 gauge hypodermic needle punctured through to allow for pressure relief. Finally, the flasks were incubated in an orbital shaker maintained at 32°C and 200 rpm. SSF of the barley mash was carried out for 72 h. Final samples were taken for the analysis of the ethanol concentration. The flasks also were weighed periodically and the weight loss due to carbon dioxide production was used to follow the progress of the ethanol production. Each experiment was performed in triplicate and, in some cases, in six replicates. At the end of the experiment, the contents of the flasks were combined and dried in an oven at 70°C. The final DDGS products were analysed for starch, β-glucan, protein, phytic acid and fibre contents. Preliminary experiments were performed in order to estimate the loss of ethanol during fermentation. In these experiments, a solution of 15% (v/v) ethanol in de-ionized water was placed in flasks with the same arrangements as described above. The results indicated that during a 72-h incubation period at 32°C and 200 rpm the loss of ethanol due to evaporation was less than 2% of the initial ethanol.

### Ethanol production without β-glucosidase

In these experiments, the process variables that were examined included the requirements of OPTIMASH™ TBG in the liquefaction and OPTIMASH™ BG in the SSF, the liquefaction time and the grain particle size. Thus, experiments were performed where OPTIMASH™ TBG was omitted in the liquefaction and OPTIMASH™ BG was omitted in the SSF, respectively. For each set of experiments, control experiments also were performed in which the base-line conditions were used. In order to study the effects of liquefaction time and particle size on ethanol production, liquefaction time of 1 h, 2 h and 3 h, and particle sizes of <1 mm and <2 mm, were used. In these experiments, the pre-liquefaction of the mash was performed under the conditions of the base-line experiment - 60°C and 1 h.

### Ethanol production with β-glucosidase and development of the EDGE process

In the first experiment performed in order to develop the EDGE process, ground barley with a particle size <1 mm was used to prepare the mash, which then was used for the ethanol production. The enzyme dosages, temperature, liquefaction time and urea concentration were the same as for those described for the base-line experiment. In the SSF, the enzyme β-glucosidase was added at a dosage of 100 μL/flask (2.44 kg enzyme per MT of dry solids). Six replicates were performed for the SSF. Another set of six SSF replicates also was performed using the conditions of the base-line experiment. In these flasks the addition of β-glucosidase was omitted. After a statistically significant improvement of ethanol production was observed for the β-glucosidase addition (see Results and discussion), experiments were performed in order to study the effects of other variables of the EDGE process on ethanol production.

#### Effect of β-glucosidase dosage

In these experiments, the β-glucosidase dosage was varied from 10 -100 μL/flask (0.244 to 2.44 kg/MT dry solids). The other process conditions were the same as in the base-line experiment.

#### Effects of liquefaction time and particle size

In these experiments, the barley mash was cooked at 60°C for 1 h and then at 90°C for 1 h, 2 h and 3 h and β-glucosidase was used at a dosage of 2.44 kg/MT dry solids. Ground barley having particle size <1 mm and <2 mm were used. The thermally stable β-glucanase, OPTIMASH™ TBG, was omitted in the pre-liquefaction.

#### Effect of pre-liquefaction temperature

Experiments were performed where the pre-liquefaction temperature was maintained at 50°C, 60°C and 70°C. In these experiments, ground barley with a particle size <1 mm was used, the pre-liquefaction time was 1 h, the liquefaction step was performed at 90°C and 2 h and the enzyme β-glucosidase was added to the SSF at 2.44 kg/MT dry solids. A separate experiment was also performed under similar conditions, except the pre-liquefaction step was omitted completely.

After the optimum conditions of the EDGE process for the particular batch of Thoroughbred used in this investigation had been established (see Results and discussion), experiments were performed to obtain DDGS samples for compositional analysis. Two batches of barley mash were prepared. Each batch then was used for SSF, which was performed in triplicate. The enzyme β-glucosidase was used at a dosage of 50 μL/flask (1.22 kg/MT total solids). Samples were taken daily from the first three flasks for ethanol analysis. In the remaining flasks only the final samples taken at 72 h were analysed. At the end of the fermentation, the entire contents of each group of three flasks were pooled together and dried in an oven at 70°C. The dry solids were analysed for ash, fibre, protein, phytic acid, starch and β-glucan.

### Analytical methods

The moisture content of whole barley kernels was obtained by drying 10 g of barley at 130°C for 20 h [18]. The moisture content of ground barley was determined by drying 2 g samples at 135°C for 2 h [19].

The ash content was determined by heating barley flour in a muffle furnace at 550°C for about 16-20 h until a light grey ash is obtained [20].

The oil content was estimated as described by Moreau *et al *[21]. Barley was ground in a Wiley mill fitted with a 20 mesh screen and 4 g samples were extracted with hexane in an Accelerated Solvent Extractor (Dionex Corporation, CA, USA). The instrument was operated at 1000 psi and a temperature of 100°C for three 10 min cycles after which the hexane extract obtained was dried under a stream of nitrogen and oil content determined gravimetrically.

For starch analysis, barley samples were ground in a cyclone mill fitted with a 0.5 mm screen (Udy, CO, USA) and the flours were analysed using a starch determination kit obtained from Megazyme International Ireland Ltd (Bray Business Park, County Wicklow, Ireland) [22]. The method was modified using a YSI 2700 Analyzer (YSI Incorporated, OH, USA) fitted with a YSI 2710 turntable for automated glucose determination of enzymatically hydrolyzed starch containing samples.

The protein content of barley flour samples was determined in accordance with standard methods [23,24]. The conversion factor used to obtain protein values for barley was 6.25 [25].

Barley β-glucan was analysed using a kit obtained from Megazyme International Ireland Ltd (Bray Business Park, County Wicklow, Ireland) according to ICC Standard Method 166 [26] and the instructions for the 'streamlined method' provided by the manufacturer. This method conforms to standard methods [27,28].

Acid detergent fibre, neutral detergent fibre (NDF) and crude fibre were determined with an Ankom 2000 fibre analyser (Ankom Technology, NY, USA) as per the methods supplied by the manufacturer. Non-fibre carbohydrate (NFC) is defined as 100% dry matter minus % crude protein (CP) minus NDF corrected for insoluble crude protein (NDICP) minus % fat and minus % ash [29]. Thus,

In order to determine the NDICP, the following method was used. Approximately 0.5 g of DDGS was weighed into an Ankom filter bag which was extracted with neutral detergent fibre reagent as instructed by the manufacturer of the Ankom Fiber Analyzer. After the extraction and drying of the filter bags the contents were weighed in order to determine the % NDF and then were re-assayed for protein using the same method as used for CP (copper catalyst/combustion method as outlined in AOAC 999.03, AACC 46-30) in order to determine the percentage of insoluble CP in the NDF fraction or NDICP. The value for % NDICP was then used to calculate the NFC value of the DDGS as shown above.

The phytic acid content of the DDGS samples was determined by high performance anion exchange chromatography at the Genencor Analytical Laboratories (CA, USA).

In order to determine the concentrations of fermentation products, samples taken from the fermentation flasks were centrifuged and the supernatants were filtered through 0.2 μm filters. Ethanol concentrations were then determined by high-performance liquid chromatography (HPLC). The system was an ISCO model 2350 using 0.5% sulphuric acid as solvent at 0.6 mL/min combined with an Aminex^® ^HPX-87H ion exclusion column (Bio-Rad Laboratories, CA, USA) operated at 60°C and an HP 1047A refractive index detector (Hewlett Packard, CA, USA). The software used for data analysis was Chrom Perfect^® ^Spirit version 4 build 17 (Justice Laboratory Software, Fife, Scotland).

### Calculation of theoretical ethanol production in SSF

Each flask contained 150 g total weight, which included 45 g dry matter (30% dry solids) and 105 g water.

The ground Thoroughbred barley contained 59.9% starch and 3.9% β-glucan. Therefore, the total fermentable carbohydrates was:

The quantity of glucose produced upon complete hydrolysis of the fermentable carbohydrates was:

Theoretical ethanol yield was:

Water consumption during hydrolysis of starch and β-glucans was:

Final liquid volume in the flask was:

Expected ethanol concentration would be:

## Results and discussion

### Ethanol production without β-glucosidase in the SSF

*Effects of OPTIMASH™ TBG in pre-liquefaction and OPTIMASH™ BG in SSF*: In the base-line experiment, the enzyme OPTIMASH™ TBG, which is more thermally stable than the other β-glucanase, OPTIMASH™ BG, was used to ensure that there would be sufficient β-glucanase activity throughout the starch liquefaction stage. The main role of OPTIMASH™ TBG is to reduce the viscosity of the mash to facilitate downstream processing in a commercial process. However, it would be interesting to determine whether the presence of this enzyme during the pre-liquefaction stage would also help to improve the ethanol production. The results summarized in Table 2 indicate there was no improvement of ethanol production by addition of OPTIMASH™ TBG in the pre-liquefaction stage. However, it should be pointed out that, in a commercial barley ethanol process, OPTIMASH™ TBG will be needed for viscosity reduction in the initial mashing stage. In fact, viscosity measurements (results not shown) indicated that, when OPTIMASH™ TBG was used, the viscosity of the mash was reduced by about one-third. High viscosity of the mash might not be important in shake-flasks where the mash was still sufficiently fluidic to allow for the distribution of the yeast and required nutrients but it would be a serious problem in a production plant, especially when downstream processing is performed. Also, in the base-line experiment, it was assumed that some OPTIMASH™ TBG could have been denatured during the 90°C liquefaction and so additional OPTIMASH™ BG was added to the SSF in order to ensure the complete hydrolysis of any remaining β-glucans. As the case of OPTIMASH™ TBG in pre-liquefaction, it would be interesting to determine if the addition of OPTIMASH™ BG in the SSF would improve ethanol production. The results obtained in experiments where this enzyme was omitted in the SSF also are compared to the base-line results in Table 2. These results show that the presence of OPTIMASH™ BG in the SSF did not improve ethanol production. This indicates that hydrolysis of all the β-glucans was effectively complete in the liquefaction step, or that activity of residual thermostable β-glucanases added during the pre-liquefaction step was capable of hydrolyzing any remaining β-glucan during SSF.

**Table 2:** The effects of OPTIMASH™ TBG in preliquefaction and OPTIMASH™ BG in simultaneous saccharification and fermentation on ethanol production in the base-line process.

Ethanol % (v/v) at 72 h fermentation time	
With OPTIMASH™ TBG in pre-liquefaction 14.82 ± 0.28*	Without OPTIMASH™ TBG in pre-liquefaction 14.65 ± 0.17*

With OPTIMASH™ BG in SSF 14.69 ± 0.18†	Without OPTIMASH™ BG in SSF 14.89 ± 0.10†

*Average of three replicates.

† Average of six replicates.

#### Effects of liquefaction time and particle size

The liquefaction time in the base-line experiment was 3 h, which may be more than necessary. We decided to determine whether it could be reduced without a negative effect on ethanol production. The results of experiments performed in order to examine the effect of shorter liquefaction times on ethanol production are summarized in Table 3. There was no improvement of final ethanol concentration when the liquefaction time was increased from 2 h to 3 h. Thus 2 h was sufficient to allow solubilization and hydrolysis of the starch to proceed to such a point that the products of this stage could be effectively converted to ethanol in the subsequent SSF. Also included in Table 3 are the results obtained with particle sizes of <1 mm and <2 mm. These results show that the two particle sizes of the ground barley used in these experiments did not have any effect on ethanol production. In other words, a reduction of the barley to particle size of 2 mm was sufficient to allow the enzymes to penetrate and effectively carry out the starch hydrolysis in this shake flask-scale process.

**Table 3:** The effect of liquefaction time (*t*_LQ_) on ethanol production in the base-line process.

	Ethanol % (v/v)	At 72 h fermentation	Time
	**t_LQ _= 1 h**	**t_LQ _= 2 h**	**t_LQ _= 3 h**

Particle size <2 mm	13.81 ± 0.22	14.45 ± 0.21	14.32 ± 0.22

Particle size <1 mm	13.69 ± 0.33	14.58 ± 0.20	14.47 ± 0.28

All values are averages of three replicates.

### Ethanol production with β-glucosidase and development of the EDGE process

It was probable that the two β-glucanases used in the experiments described previously hydrolyzed the β-glucans to both glucose and glucose oligomers, which were not fermentable by *S. cerevisiae*, similar to the action of other β-glucanases [14]. Therefore, experiments were performed to determine whether the addition of β-glucosidase could increase ethanol production by hydrolyzing the glucose oligomers to glucose, which is readily fermentable by the yeast to ethanol. The experimental procedure was described above. The 72 h ethanol concentrations obtained without β-glucosidase and with this enzyme added at 2.44 kg/MT dry solids were 14.86 ± 0.19% (v/v) and 15.32 ± 0.19% (v/v), respectively. These results are averages of six replicates in each case. The *t*-test also was performed on the two data sets and the probability obtained was 0.0015, which indicates that the two data sets are statistically different at 95% confidence level.

As a result of the increase in ethanol production by the addition of the enzyme β-glucosidase we designated our new process the EDGE process. This increase is equivalent to a 3.1% improvement. Thus, in an ethanol plant producing 50 million gallons per year (MGY) using the base-line process, an additional 1.5 MGY of ethanol can be expected if the EDGE process is used. It should be pointed out that these results were obtained with a particular batch of Thoroughbred. Although there is no reason to doubt that the EDGE process will not be applicable to other barley feedstocks, experiments will have to be performed in order to determine the actual improvement on ethanol production.

The effect of β-glucosidase at lower dosages on ethanol production also was studied. These results are summarized in Table 4. The results obtained with no addition of β-glucosidase and those obtained with 2.44 kg β-glucosidase per MT dry solids are also are included.

**Table 4:** The effect of β-glucosidase dosages on ethanol production in the enhanced dry grind enzymatic process.

B-Glucosidase	Dosage		
**μL/flask**	**Kg/MT dry solids**	**Ethanol % (v/v)**	**Maltose/cellobiose** **(g/L)**

0	0	14.86 ± 0.19	5.83 ± 0.24

10	0.244	14.46 ± 0.16	5.06 ± 0.10

25	0.61	14.82 ± 0.20	3.20 ± 0.12

50	1.22	15.15 ± 0.09	1.34 ± 0.05

75	1.83	15.35 ± 0.07	1.40 ± 0.10

100	2.44	15.32 ± 0.19	1.09 ± 0.05

The results indicate that β-glucosidase dosages of 1.22 kg/MT dry solids, or higher, were needed to obtain an observable improvement of ethanol production. These results also show that increasing the β-glucosidase dosages above 1.22 kg/MT dry solids still improved ethanol production, albeit by relatively small margins. When the enzyme dosage was increased from 1.22 kg/MT dry solids to 1.83 kg/MT dry solids ethanol production was increased by only 1.3%. A further increase of the enzyme dosage did not result in any additional improvement of ethanol production.

The concentrations of disaccharides in the 72 h samples from experiments where various β-glucosidase dosages were used decreased with the increases in β-glucosidase dosages. Although the HPLC disaccharide peak measures both cellobiose and maltose together, β-glucosidase does not act on the α-linkage in maltose. Thus, the gradual decrease in the concentrations with increasing β-glucosidase dosages was a clear indication of cellobiose hydrolysis to glucose by this enzyme.

### Optimization of the EDGE process

#### Effects of liquefaction time and particle size

The results of the experiments performed to examine the effect of liquefaction time and particle size are summarized in Table 5. The results show that liquefaction time of 2 h was sufficient and the particle sizes of the ground barley used in the experiments (< 1 mm and < 2 mm) did not have any effect on ethanol production, which also was observed previously in the base-line process.

**Table 5:** The effects of liquefaction time (*t*_LQ_) and particle size on ethanol production in the enhanced dry grind enzymatic process.

	Ethanol %	(v/v) at 72 h	
	**t_LQ _= 1 h**	**t_LQ _= 2 h**	**t_LQ _= 3 h**

Particle size <2 mm	14.02 ± 0.10	14.71 ± 0.06	14.36 ± 0.04

Particle size <1 mm	14.02 ± 0.19	15.04 ± 0.15	14.84 ± 0.02

#### Effect of pre-liquefaction temperature

The purpose of the pre-liquefaction step is to gelatinize the starch to facilitate hydrolysis by the thermostable α-amylase in the subsequent liquefaction step. The pre-liquefaction step in the conventional process was performed at 60°C and 1 h. We examined the effect of pre-liquefaction temperature on ethanol production from the batch of Thoroughbred used in this investigation to see if other temperatures gave improved benefits. Thus, experiments were performed where the pre-liquefaction temperature was maintained at 50°C, 60°C and 70°C as described previously. These temperatures were chosen to cover the gelatinization temperature range of barley, which was reported as 53°C to 70°C [30,31]. The lowest temperature of 50°C was also chosen because it has been reported that heating of barley starch below this temperature did not increase the accessibility of the starch toward the enzyme α-amylase from *Bacillus licheniformis *[32]. The results of these experiments are summarized in Table 6.

**Table 6:** The effect of pre-liquefaction temperature (*T*_PLQ_) on ethanol production in the enhanced dry grind enzymatic process.

	Ethanol % (v/v) at	At 72 h	
***T*_PLQ _= 50°C**	***T*_PLQ _= 60°C**	***T*_PLQ _= 70°C**	**No pre-liquefaction**

15.40 ± 0.06	15.31 ± 0.12	15.17 ± 0.21	15.49 ± 0.03

The results indicate that the different pre-liquefaction temperatures did not have an effect on ethanol production. Even when the pre-liquefaction step was eliminated, the same ethanol yield was observed. In the experiment where the pre-liquefaction step was omitted, it took about 30 min for the mash to reach the liquefaction temperature (90°C). In that case, the 30 min preheating period in effect served as the pre-liquefaction treatment of the mash.

#### Ethanol production under optimum shake-flask conditions

Based on the results obtained the following conditions are chosen for the shakeflask-scale EDGE process for Thoroughbred barley used in this work:

a. No pre-liquefaction step

b. Liquefaction time of 2 h at 90°C

c. No addition of OPTIMASH™ TBG in the liquefaction

d. No addition of OPTIMASH™ BG in the SSF

e. Dosage of OPTIMASH™ BG in liquefaction: 0.13 kg/ton dry solids

f. Dosage of SPEZYME^® ^Xtra in liquefaction: 0.30 kg/ton dry solids

g. Dosage of FERMENZYME^® ^L-400 in SSF: 0.65 kg/MT dry solids

h. Dosage of β-Glucosidase added during SSF: 1.22 kg/MT dry solids.

**Figure 1:**
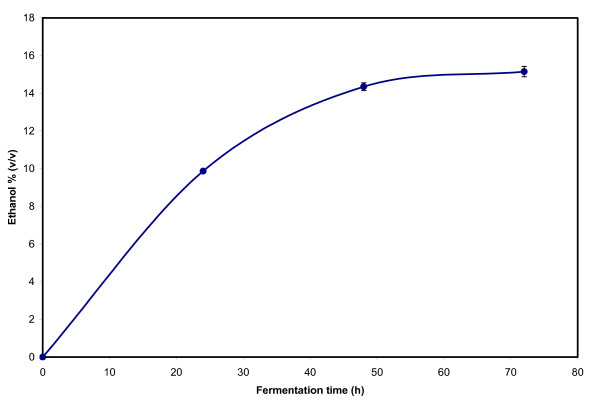
**Ethanol concentration profiles in the EDGE process using the established optimum conditions**. The error bars show the standard deviations of the measured values.

Experiments were performed under these conditions in order to determine the ethanol yield and also to obtain the DDGS for compositional analysis. The ethanol concentration profiles in the first three flasks are shown in Figure 1. At 48 h already 95% of the total ethanol had been produced. The SSF, therefore, would not need to be carried out for 72 h. A fermentation time of 55 h to 60 h should be sufficient. While these conditions are optimized for shake-flask studies, they are expected to be useful for commercial scale fermentations, although, as previously noted, the inclusion of pre-incubation steps with added enzymes will be necessary for reducing viscosity enough for pumping and handling the mash.

The final ethanol concentration averaged for all six flasks was 15.07 ± 0.21% (v/v). This is equivalent to 89.4% of the theoretical ethanol yield based on both starch and β-glucan contents of the barley used in the experiment. A yield less than the theoretical value was obtained because the carbon source glucose was also used for yeast cell synthesis and the production of other minor products such as glycerol, lactic acid, succinic acid and acetic acid. All of these co-products of ethanol were observed during HPLC analysis of the samples. The concentrations of glycerol, lactic acid, succinic acid and acetic acid were 9.17 ± 0.21, 0.67 ± 0.16, 2.27 ± 0.20, and 0.56 ± 0.11 g/L, respectively. Even for glycerol, which was the by-product that accumulated in the largest amounts, the final concentrations were still much lower than those of ethanol. These results were expected for *S. cerevisiae*, which has been known as one of the most effective ethanol-producing organisms. The ethanol yield by the EDGE process using the conditions described is 402 L/MT (dry basis) or 2.17 gallons/bushel if we assume one bushel contains 53 lb barley and the moisture content of the barley is 15%. The ethanol yield in the base-line process calculated from the results described previously is 395 L/MT dry solids or 2.13 gallons/53-lb bushel at 15% moisture. Thus, in a plant producing 50 MGY of ethanol by the base-line process, which requires 23,474,178 bushels of barley, use of the EDGE process would reduce the quantity of barley needed to 23,041,475 bushels. Assuming a barley cost of US$2.50 per bushel, the use of the EDGE process would result in annual savings of US$1.08 million in barley cost. Again, these results are specifically applicable to the particular batch of Thoroughbred used in this investigation. Experiments will have to be performed for other barley feedstocks in order to determine the corresponding ethanol yields.

The results of the compositional analysis of the DDGS obtained in the aforementioned experiments using the EDGE process are summarized in Table 7.

**Table 7:** Composition of the DDGS obtained in the EDGE process using the established optimum conditions.

Component	% (dry basis)
NDF	39.36

Protein	21.75

NDI CP	4.25

Fat	4.53

Ash	5.71

Phytic acid	1.6

NFC	32.9

Starch	1.64

B - GLucans	0.2

Mass balance: NFC + NDF + protein + fat + ash = 104.25%.

NDF, neutral detergent fibre; NDICP, neutral detergent fibre corrected for insoluble protein; NFC, non-fibre carbohydrate.

The results in Table 7 show that the β-glucan content of the DDGS was extremely low. The β-glucan conversion was calculated to be greater than 99% of the original β-glucan. Starch conversion also was calculated to be 99%. The protein and NDF contents of the barley DDGS are both slightly lower than those in corn DDGS, which are reported as 30.92 and 44.73%, respectively [33], but still are at levels suitable for use in animal feeds. The phytic acid content was 1.60% or 16 g/kg dry matter, which is considerably higher than the values reported for some animal feeds, which are 8.85, 10.80 and 9.02 mg/g dry matter for pigs, sows and hen feeds, respectively [34]. However, since DDGS always make up just a fraction of an animal feed ration, this level may be acceptable. The modification of process conditions or the addition of phytase could potentially be used to further reduce the levels of phytic acid in the DDGS product if necessary.

## Conclusions

A significant improvement in the production of fuel ethanol from Thoroughbred hulled winter barley has been demonstrated. The combined use of two enzymes, β-glucanase, which hydrolyzed the soluble β-glucans to oligosaccharides and, consequently, reduced the high viscosity of the mash, and β-glucosidase, which converted the non-fermentable oligosaccharides formed in the hydrolysis of β-glucans to the fermentable sugar glucose, allowed ethanol to be produced at 30% total dry solids to reach 15% v/v. Under optimum conditions of the newly developed EDGE process, an ethanol yield of 402 L/MT (dry basis) or 2.17 gallons/bushel of barley was achieved on the shake-flask scale. The DDGS co-product with extremely low β-glucan contents should be suitable as an ingredient in both ruminant and mono-gastric animal feeds.

## Abbreviations

CP: crude protein; DDGS: distillers dried grains with solubles; EDGE: enhanced dry grind enzymatic; HPLC: high-performance liquid chromatography; MGY: million gallons per year; NDF: neutral detergent fibre; NDICP: neutral detergent insoluble crude protein; NFC: non-fibre carbohydrate; SSF: simultaneous saccharification and fermentation.

## Competing interests

The authors declare that they have no competing interests.

## Authors' contributions

NPN designed, coordinated the experimental work, analysed the results and drafted the manuscript. GS contributed to the design and performed the experiments. KBH, DBJ, JS, ML and GKJ contributed to the original concept and advised on the design and progress of the experimentation. MK developed several analytical procedures and coordinated the analytical work. All authors critically reviewed the draft and approved the manuscript in its final form.
